# Are Biodegradable Calcium Sulfate Antibiotic Beads Effective and Safe Adjuvants for Diabetic Foot Osteomyelitis?

**DOI:** 10.7759/cureus.52444

**Published:** 2024-01-17

**Authors:** Talal Alkayali, Dominick Casciato, Jacob Wynes, Joel Chua, James B Doub

**Affiliations:** 1 Infectious Disease, University of Maryland, Baltimore, USA; 2 Podiatry, University of Maryland, Baltimore, USA; 3 Infectious Disease, University of Maryland Medical Center, Baltimore, USA

**Keywords:** diabetic foot ulcer management, diabetic foot infections, diabetes, calcium sulfate, antibiotic beads, osteomyelitis treatment

## Abstract

Introduction: Diabetic foot osteomyelitis (DFO) is a highly morbid condition that commonly affects diabetic patients. Biodegradable calcium-sulfate antibiotic beads (CaSO4) are theoretical adjuvant agents to reduce morbidity in DFO. However, there is a paucity of research on the safety and effectiveness of CaSO4 beads in DFO. Therefore, the purpose of this study was to assess the safety and effectiveness of CaSO4 beads in different DFO locations.

Methods: We conducted a retrospective cohort study between January 1, 2015 and January 1, 2022 of patients with DFO who underwent surgical intervention and adjuvant CaSO4 beads placement. The location of DFO was determined based on the forefoot, midfoot, or hindfoot locations. Outcomes measured were ulcer-free time points of three and six months as well as recurrence of DFO at 12 months. Safety was also evaluated with incidences of acute kidney injury, wound drainage, and hypercalcemia.

Results: Forty-five cases were included. Of these, only 9/45 (20%) and 13/45 (29%) were ulcer-free at three months and six months, respectively. DFO recurred in 19/45 (42%) patients. Safety outcomes were significant for wound drainage (62%) and acute kidney injury (9%). Stratifying according to the location of DFO showed no statistically significant difference in outcomes.

Conclusion: In this cohort study, adjuvant CaSO4 beads showed high rates of ulcer persistence and DFO recurrence. Given the limited benefits seen here and the potential for high rates of wound drainage, the use of adjuvant CaSO4 beads should be used cautiously until a multicenter randomized clinical trial is conducted to definitely evaluate the safety and effectiveness of CaSO4 beads in DFO.

## Introduction

The global prevalence of diabetes continues to increase with well over 450 million people afflicted with diabetes [1]. Unfortunately, approximately one-fifth of diabetic patients will require hospitalization for a diabetic foot infection [2]. These infections are devastating complications that are associated with significant morbidity and enormous financial repercussions. Diabetic foot infections typically start with ulcers that require multidisciplinary treatment approaches to prevent the progression to osteomyelitis [3]. Once diabetic foot osteomyelitis (DFO) develops aggressive treatments are needed to thwart the need for amputations, but amputations are often required to cure the infection while also preserving functionality of the foot [3,4]. However, amputations are notorious to be associated with poor wound healing, further ulcerations, and the need for more proximal amputations [5,6]. Consequently, novel therapeutics are needed to aid in sterilization, and wound healing while also preventing infection recurrence.

One such therapeutic is biodegradable calcium sulfate antibiotic-impregnated beads (CaSO4 beads) [6]. These beads slowly elute antibiotics and calcium to local tissues and surgical dead spaces [7]. This allows for high local concentrations of antibiotics to be well above the minimal inhibitory concentration thus adding in sterilization of these local environments. As well, the release of calcium aids in wound healing. The use of calcium sulfate beads has been evaluated in small podiatric studies, but these studies often have heterogeneous patient populations and frequently do not evaluate for potential side effects of these agents in DFO [8-10]. Moreover, there is a paucity of research evaluating the long-term effectiveness of these beads in DFO with respect to the placement of these beads in different locations of the foot [8-10]. Consequently, the aims of this study were to compare the long-term outcomes and safety of CaSO4 beads for DFO when these beads were placed in the forefoot, midfoot, or hindfoot.

## Materials and methods

Patients and CaSO4 beads

We conducted a retrospective cohort study (approved by the University of Maryland Internal Review Board HP-00099147) on the use of adjuvant CaSO4 beads for the treatment of DFO at our academic clinical center. Patients were identified using current procedural terminology (CPT) codes (11981, 20700). Patients 18 years and older with a known history of DFO and who had undergone CaSO4 beads placement as an adjuvant therapy between January 2015 to January 2022 were included. Patients with no records of follow-up evaluation at 12 months were excluded. Data extracted from the electronic medical record included demographics, comorbidities, location of DFO, causative pathogens, antibiotic treatments, safety, and outcomes. Locations of DFO were divided into forefoot, midfoot, and hindfoot. If there was an overlap of the site of infection, the case would be classified as the more proximal site.

Antibiotics used in the CaSO4 beads were vancomycin, tobramycin, gentamycin, linezolid, and meropenem. The antibiotics were chosen based on their broad-spectrum activity and reported allergies by patients. The amount of CaSO4 beads used in our cases was 10mL except for one case that has 20mL of CaSO4 beads used. The decision to place CaSO4 beads was determined by the podiatry surgeon performing the surgery. All patients received concomitant systemic antibiotics either intravenous or oral therapy for six weeks.

Variables and outcome measures

The outcomes measured were ulcer-free at three- and six-month follow-up as well as recurrence of osteomyelitis up to 12 months from index surgery. Safety outcomes measured included the development of acute kidney injury (AKI) within 30 days of exposure to beads (defined by an increase in serum creatinine by 1.5-fold compared to baseline), hypercalcemia >10.5 mg/dL within 30 days of exposure to beads, and drainage from the surgical site at any follow-up appointment up to three months after exposure to beads. Analysis of outcomes was conducted using descriptive statistics and the Chi-square test was used to compare proportions with a p-value less than 0.05 represent statistical significance.

## Results

**Figure 1:**
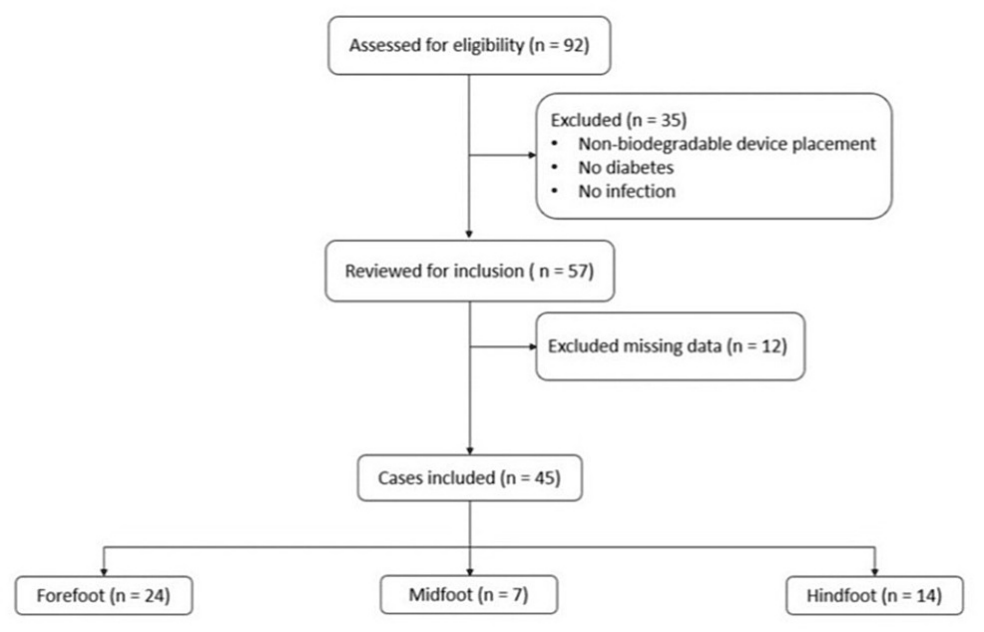
Flow chart of patients included in cohort study

A total of 92 cases of DFO who underwent antibiotic implants were identified, of whom 57 had DFO that underwent calcium sulfate antibiotic beads placement, the remaining 35 cases were excluded for not meeting criteria (no infection, no diabetes mellitus, or placement of non-biodegradable antibiotic material) (Figure 1). Twelve additional cases were excluded due to missing data or no follow-up, resulting in 45 total cases for analysis. Locations of DFO included forefoot (n=24, 53.3%), midfoot (n=seven, 15.6%), and hindfoot (n=14, 31.1%), one case had overlap between forefoot and midfoot that was added to the midfoot cohort, and one case had overlap between midfoot and hindfoot that was added to hindfoot cohort for ease of stratification.

Baseline characteristics of the 45 patients included in the analyses are summarized in Table 1. Majority were male (n=31, 69%) and Black or African American (n=29, 64.4%) (Table 1). All patients had diabetes mellitus and 47% (n=21) had peripheral vascular disease (PVD). An additional 36% (n=16) had chronic kidney disease (CKD), of which, four were receiving dialysis. A total of 22% (n=10) of the cohort was immunocompromised in which 11% (n=five) had HIV.

**Table 1:** Cohort demographics ^1^Immunocompromised included two kidney transplant, one liver transplant, one chronic myeloid leukemia, one plaque psoriasis on immunotherapy.

Total cases, n	45
Age, mean (range)	58 (31-78)
Male, n (%)	31 (69%)
Race, n (%)	
Black	29 (64.4%)
White	15 (33.3%)
Asian	1 (2.2%)
Ethnicity, n (%)	
Non-Hispanic	45 (100%)
BMI n (%)	
18.5-24.9, n (%)	4 (8.9%)
25-29.9, n (%)	20 (44.4%)
30-34.9, n (%)	10 (22.2%)
>35, n (%)	11 (24.4%)
Comorbidities	
Diabetes mellitus, n (%)	45 (100%)
Peripheral vascular disease, n (%)	21 (47%)
Chronic kidney disease, n (%)	16 (36%)
Tobacco use, n (%)	14 (31%)
Immunocompromised^1^, n (%)	5 (11%)
HIV, n (%	5 (11%)

Overall, only nine patients (20%) achieved ulcer free outcomes at three months and 13 patients (29%) were ulcer free at six months. Furthermore, 19 patients (42%) had recurrence of osteomyelitis at 12 months. When stratified based on location (Table 2) there was no significant differences between outcomes. There were also no statistically significant differences in outcomes between patients with and without PVD (Table 3).

**Table 2:** Location of infection and outcome

	Overall (N=45)	Forefoot (N=24)	Midfoot (N=7)	Hindfoot (N=14)	P-values
Ulcer free 3 months	9 (20%)	6 (25%)	1 (14%)	2 (14%)	P = 0.5464
Ulcer free 6 months	13 (29%)	8 (33%)	2 (29%)	3 (21%)	P = 0.6915
Recurrence of osteomyelitis in 12 months	19 (42%)	10 (44%)	4 (57%)	5 (36%)	P = 0.5508

**Table 3:** Evaluation of diabetic foot osteomyelitis and peripheral vascular disease

	Patients with PVD	Patients with no PVD	P-value
Ulcer free 3 months	2/21 (9%)	7/24 (29%)	P = 0.0959
Ulcer free 6 months	4/21 (19%)	9/24 (37%)	P = 0.1874
Recurrence of Osteomyelitis	8/21 (38%)	11/24 (46%)	P = 0.5920

Table 4 shows the safety outcome measured. To note, wound drainage was the most noted side effect compromising 28 cases (62%). AKI was seen in four cases and no hypercalcemia was observed in this cohort. With regards to safety measures, there were no statistically significant difference groups when stratified based on location of CaSO4 bead placement.

**Table 4:** Safety outcome Acute kidney injury is defined by an increase in serum creatinine at least by a 50% from baseline within seven days. Hypercalcemia is defined as serum calcium >10.5mg/dL.

Location	Overall (N=45)	Forefoot (N=24)	Midfoot (N=7)	Hindfoot (N=14)	P-values
Wound drainage	28 (62%)	14 (58%)	6 (86%)	8 (57%)	P = 0.3650
Acute kidney injury	4 (9%)	2 (8%)	1 (14%)	1 (7%)	P = 0.6370
Hypercalcemia	0 (0 %)	0 (0%)	0 (0%)	0 (0%)	P = 1.0000

## Discussion

DFO is a highly morbid and costly complication for patients living with diabetes. Despite appropriate surgical intervention and medical management, 40% (n=18) of patients develop recurrence of osteomyelitis which requires further surgical interventions and proximal amputations [11]. Bioabsorbable CaSO4 beads have been used for bone, soft tissue, and even vascular graft infections with varied success [5,12,13]. However, only a few published studies have evaluated the outcome of calcium sulfate beads for diabetic foot infections most of which have been case series with success rates ranging from 66% to 92% [8-10,14,15]. Yet those studies have been biased by short outcomes measured (less than three months) and by excluding patients with PVD. Consequently, the ability of adjuvant CaSO4 beads to prevent long-term recurrence of osteomyelitis is unknown as is the ability to aid in the treatment of patients with PVD which is a common comorbidity in these patients. 

Therefore, we evaluated the ability of CaSO4 beads to prevent DFO recurrence and achieve ulcer-free outcomes at prolonged time points as well as in patients with PVD. This therefore provided a more realistic patient cohort with clinically relevant outcome measurements. As seen in this cohort, despite the addition of biodegradable CaSO4, 80% (n=36) had persistent ulcers at three months, 71% (n=32) at six months, and 42% (n=19) had a recurrence of DFO at their 12 months follow-up. There was not a statistically significance difference between outcomes associated with the different foot locations nor in patients with or without PVD. The lack of overall reduction in recurrence in osteomyelitis when compared to historical recurrence rates (approximately 40%) suggests that DFO is a complex disease requiring more than just high concentrations of locally administered antibiotics and the effectiveness of CaSO4 beads in DFO may be limited. 

Moreover, in our cohort, we observed high rates of wound drainage in 28 patients (62.2%) associated with ulcer persistence at three and six months. This raises concern that the addition of CaSO4 beads in podiatric surgery may be correlated with this finding. Wound drainage with CaSO4 beads has been one of the most frequently reported adverse events that are not associated with worsening infections but rather from the beads themselves [16,17]. Some reports have documented wound drainage to be as high as 33.3% with the use of CaSO4 beads [16-19]. In our study, wound drainage from the surgical site was much higher than what other orthopedic studies have reported [16-19]. A possible explanation for these findings is that the foot anatomy and subsequent bead placement is much more superficial given limited muscular, and subcutaneous tissues compared to arthroplasty surgery where the beads can be placed in deeper soft tissues. This results in local inflammatory changes being more pronounced in outward symptomology with wound drainage than would be seen if placed in deeper surgical dead spaces. While this will need to be undoubtedly be evaluated in further studies, at the present time it is important for clinicians to be cognizant of DFO and the associated clinical ramifications.

Also associated with the limited soft tissues in the foot are the restricted amounts of CaSO4 beads that can be used in these locations. In our cohort, all except one case had only 10 mL of CaSO4 beads placed in the soft tissues given the limited space that foot wounds can typically accommodate. This is important because, in each 10 mL of CaSO4, the amount of elemental calcium constitutes 5.7 grams. Thus, the low amount of elemental calcium that patients in this cohort were exposed to was less than 40 mL of CaSO4 which the Food and Drug Administration warns can then be associated with transient hypercalcemia [6,20,21]. It was therefore not surprising that we saw no case of hypercalcemia. Moreover, recommendations are to use 1,000 mg of vancomycin, 240 mg tobramycin, or 240 mg gentamycin in each 10 mL of CaSO4, which then elutes from the beads over weeks [21,22]. Consequently, when low volumes of CaSO4 are used, patients are exposed to only small concentrations of these potentially nephrotoxic antibiotics thereby reducing the risk for adverse renal-associated events. In our cohort, four patients had AKI, but the AKI in these cases was thought to be secondary to underlying kidney disease and systemic antibiotics were administered given that AKI recovered and CaSO4 beads were not removed.

While this study highlights a potential side effect of CaSO4 beads in DFO and shows limited effectiveness in DFO, this study does have several limitations. Overall, the small sample size and lack of a control group limit comparisons, but the findings reinforce that DFO is a complex infectious condition that requires more than high local concentrations of antibiotics. Also, the majority of the cases were observed during the SARS-CoV-2 pandemic which to some extent did affect in-person follow-up with the surgeon, wound care, and higher percentage of noncompliance from the patients either by weight bearing, lifestyle modification as smoking cessation and strict blood glucose control which could have affected wound healing as independent factors. However, DFO requires multidisciplinary care, and these factors occur even despite the COVID-19 pandemic therefore mitigating this bias.

## Conclusions

In conclusion, CaSO4 beads are a theoretical adjuvant in the treatment of DFO that provides high local concentrations of antibiotics to the site of infection. Unfortunately, the overall effectiveness of CaSO4 beads has not been robustly studied with long-term outcomes nor in patients with PVD. Here we show that with long-term follow-up there were high rates of poor wound healing and DFO recurrence. Moreover, we observed a high rate of wound drainage which most likely is due to the placement of CaSO4 beads near the skin, but prospective studies are needed to confirm this finding. Consequently, this study did not replicate previous studies' effectiveness. Thus, the use of CaSO4 beads in the treatment of DFO should be used cautiously until a multicenter randomized clinical trial is conducted to definitely determine the safety and efficacy of CaSO4 beads in DFO.
